# Uncovering trajectories of personality functioning in adolescence and their associations with baseline psychopathology

**DOI:** 10.3389/fpsyt.2026.1751455

**Published:** 2026-02-24

**Authors:** Elena Gaudiešiūtė, Carla Sharp, Gabrielė Skabeikytė-Norkienė, Rasa Barkauskienė

**Affiliations:** 1 Institute of Psychology, Vilnius University, Vilnius, Lithuania; 2 Department of Psychology, University of Houston, Houston, TX, United States

**Keywords:** adolescence, developmental trajectories, DSM-5 AMPD, ICD-11, LoPF-Q 12-18, personality functioning, psychopathology

## Abstract

As the conceptualization of personality disorders shifts from a categorical to a dimensional assessment, understanding the trajectory of personality functioning in adolescence becomes crucial. Thus, this study aimed to examine distinct trajectories of personality functioning over a two-year period in a community sample of adolescents, while identifying associated factors. A three-wave longitudinal study with a one-year interval between assessments was conducted from 2021 to 2023 with Lithuanian adolescents aged 11-18 (N = 855; M = 14.44, SD = 1.60; 62.5% female, 37.5% male) from a community sample. Personality pathology was assessed using the Levels of Personality Functioning Questionnaire (T1-T3). Associated factors included measures of internalizing and externalizing problems, victimization, and self-harm. Latent class growth analysis revealed four distinct trajectories of personality functioning: adaptive and declining, as well as three stable trajectories categorized as normative, slightly impaired, and significantly impaired. Membership in the impairment groups was associated with age and sex, as well as internalizing and attention-deficit/hyperactivity problems at the first measurement. Our study presents emerging evidence of noticeable individual variability in the course of personality functioning during adolescence and informs about associations with general psychopathological symptoms.

## Introduction

The introduction of DSM-5 Alternative Model for Personality Disorders (AMPD) marked a conceptual shift in personality disorder (PD) understanding (1) and acted as a catalyst for research, incorporating dimensional models of personality pathology. The transition from a categorical to a dimensional assessment of personality disorders, moving away from symptom categorization, facilitates an emphasis on the intrapsychic aspects of personality when conceptualizing personality disorders (2). These aspects of personality are represented in the DSM-5 AMPD Criterion A (Level of Personality Functioning; LPF), which assesses an individual’s self and interpersonal functioning, including their identity, self-direction, empathy, and intimacy. LPF offers a more developmentally sensitive marker of personality processes going awry, which may signify an emerging personality disorder among adolescents (2, 3). During adolescence, youth test various aspects of their personality as they navigate new developmental challenges, including managing diverse roles, making firm commitments, and setting appropriate goals, be that academic, career-related, or personal, and adhering to them (4). These important developmental tasks were cogently articulated decades ago by Erikson (1968) (5), who emphasized that a major developmental task of adolescence is that of resolving identity and role confusion, a developmental process that extends into adulthood. Much research has supported Erikson’s theory and most recently, difficulties in achieving this developmental milestone in self and identity have been integrated as primary features of personality disorder in the new DSM-5 AMPD (1) and ICD-11 (6). It is during adolescence that an internalized and evolving life narrative emerges, aiding in the interpretation of past experiences and in creating expectations for the future, thereby contributing to the understanding of one’s identity (7). As a result, adolescents become prepared to construct a coherent and integrated life story that imparts meaning, purpose, and temporal continuity to their lives, which is the basis of an integrated sense of self, which, when interrupted, essentially defines the onset of personality disorder (8, 9). Dimensional models of PD allow for the opportunity to identify and capture impairments that may not reach the threshold of disorder but still warrant intervention (10).

Studying the course of normal and problematic personality functioning is important for understanding the underlying processes and factors contributing to increases in impairments. This is because personality pathology in adolescence is thought to be strongly associated with negative psychosocial outcomes, including low educational qualifications, social isolation, NEET (not in education, employment, or training), and low life satisfaction, among others (11). It is thought that, though the diagnostic stability of PD in adolescence and adulthood is relatively low (12), the negative psychosocial impact associated with personality disorder remains quite stable (13, 14). Given the negative outcomes linked to personality pathology, there is an ongoing necessity to pinpoint the factors contributing to the development and emergence of PD in adolescence.

Most information about the course of personality disorder is derived from studies utilizing the categorical approach to personality pathology, specifically focusing on borderline personality disorder (BPD). Systematic reviews aimed to identify the risk factors associated with the onset (15), the course (16), and poor prognosis of BPD (17) show that various categories of factors, such as genetic, biological, interpersonal, person-related, and their interactions, are associated with the onset and progression of BPD. Winsper and colleagues (2016) conducted a systematic review of the psychopathological and etiological validity of youth BPD, concluding that it is a valid construct sharing etiological features with the adult disorder, mirroring early reviews of the literature (18, 19). Confirmed factors found associated with youth BPD included mood and anxiety disorders, substance use, eating disorders, suicidal ideation and self-harm, and childhood maltreatment. Subsequently, Skabeikyte and Barkauskiene (2021) conducted a systematic review investigating factors associated with changes in the course of BPD during adolescence, identifying similar correlates, including childhood psychopathology, such as oppositional behavior, hyperactivity/impulsivity (ADHD), alcohol use disorder (AUD), depressive disorder (MDD), somatization, anxiety problems, and victimization (16).

Despite this research, our understanding of the trajectory of change in PD symptoms, whether defined categorically or specifically dimensionally, remains notably scarce in adolescents. A systematic review of the course of BPD in adolescence found that a declining trajectory of BPD features was observed in the majority of the analyzed studies; however, several studies identified subgroups of adolescents whose BPD features persisted or even increased during adolescence in both clinical and community samples (16). To date, we have identified several studies that have explored the latent trajectory of changes in personality functioning and impairment. In the study by Eggermont et al. (20), four trajectories were identified, representing low, normative, at-risk groups, and, mirroring the BPD research (21), a group of adolescents who reported high levels of impairment which increased over time. Eggermont et al. found the group with the highest PF impairments to have the highest initial levels of depressive symptoms, eating disorder symptoms, and substance use. In another study of intra-individual change over time, Bogaerts and colleagues (22) evaluated a key component of LPF, namely identity impairments. They identified a four-class solution, which depicted adaptive, increasing, and decreasing levels of consolidated identity, as well as the diffused state of identity formation, which was associated with higher levels of depression and BPD, and lower levels of self-esteem and resilience. Emergent findings indicate that personality pathology measured dimensionally may surpass categorical measurement in predicting psychosocial disability in adolescents (13), and it might be developmentally more sensitive in detecting emerging personality pathology (2). Yet, further research is needed to understand the associations between the aforementioned predictors of personality pathology with AMPD-defined personality functioning, particularly in adolescence, when personality pathology is emerging. These studies provide emerging evidence of changes in the course of personality functioning during adolescence; however, replication of these findings is needed.

With the change of conceptualization in PD towards a dimensional approach, it is important to understand the course of personality functioning in adolescence and investigate the factors associated with personality impairments measured dimensionally as they emerge during adolescence. The current study aims to investigate the presence of distinct latent trajectories of personality functioning over a two-year period in a community-based sample of adolescents. Based on the aforementioned findings, we expected to find at least two or more trajectories of personality functioning, with one group presenting with elevated and stable scores. Additionally, building upon systematic reviews of BPD in both adolescent and adult populations, we selected factors associated with the course of PD in adolescence as we aim to examine whether various psychopathological symptoms, self-harm, and victimization at baseline differentiate empirically derived PF trajectories.

## Methods

### Participants and procedure

The data presented are part of a large-scale longitudinal study in Lithuania, which explores various aspects of adolescent personality and psychosocial functioning, and received approval from the Psychological Research Ethics Committee at Vilnius University. It was carried out at three annual measurement points from September 2021 to November 2023.

A non-probability quota sampling strategy was used, with *a priori* quotas for age (11–18 years; early, middle, and late adolescence) and geographic area (urban, town, rural), chosen to reflect key developmental stages and school contexts in Lithuania. Public schools were purposively selected, and quotas were largely achieved, with minor deviations due to school participation and parental consent. Invitations and parental consent forms were sent to pupils through selected schools, and only adolescents with written parental consent took part in the study. Participants were informed about their right to withdraw at any time. Trained research assistants conducted the study during school hours in small pupil groups, where they filled out questionnaires. Participants didn’t receive any participant reimbursement for participation in the study. If students changed schools or cities at Times 2 and 3, they were contacted individually, and a questionnaire was mailed to their homes. The initial sample included 855 adolescents aged 11-18 (M = 14.44, SD = 1.60), with a gender distribution of 62.5% female and 37.5% male. Attrition analyses showed no significant differences between participants retained at T3 and those lost to follow-up in baseline age (t(852) = −1.28, p = .202) or gender (χ²(1) = 0.004, p = .952); dropouts showed higher baseline LoPF scores, but this difference was not statistically significant (t(823) = −1.75, p = .081). Enrollment originated from public schools located in various cities (37.2%), towns (40.9%), and rural areas (21.9%) in Lithuania. More than half of the participants (66.5%) reported that their parents were married, while others reported parental divorce (18.5%) or categorized family relationship status as “other” (10.90%). At Time 2, 806 (94,3%) students participated in the study, and at Time 3, 750 (87,7%) students participated. In the present study, data from all three waves were used for the Levels of Personality Functioning Questionnaire (LoPF-Q 12-18), whereas data from all other questionnaires were used solely from T1.

### Measures

#### Levels of Personality Functioning Questionnaire

Personality pathology was assessed using the culturally adapted Lithuanian version of the DSM-5-based Levels of Personality Functioning Questionnaire (LoPF-Q 12-18) (23), which was developed based on the original instrument by Goth et al. (24). This 97-item self-report instrument employs a 5-step response format (0 = no to 4 = yes), with higher scores indicating more severe impairment in personality functioning and an increased risk for a current personality disorder. This study employed an overall score for personality functioning, calculated by summing the scales of the questionnaire, as recent research suggests that a unidimensional structure provides a more suitable fit (25, 26). The total LoPF-Q 12–18 score demonstrated excellent internal consistency (Cronbach’s α at T1 = 0.97, at T2 = 0.96, and at T3 = 0.92).

#### Internalizing and externalizing problems

The assessment of psychopathological symptoms in adolescents relied on the Youth Self Report (YSR 11-18) (27). For this study, we utilized an adapted and standardized Lithuanian version of the scale (28). A set of 112 statements addressing internalizing and externalizing problems was evaluated on a 3-point scale: 0 for ‘not true’, 1 for ‘partly or sometimes true’, and 2 for ‘often or very often true’ over the last 6 months. These statements are categorized into diagnostic criteria-based scales (DSM-oriented scales), covering affective, anxiety, somatic, attention deficit/hyperactivity, oppositional defiant, and conduct problems. The total scale score is derived by summing the scores of the individual statements that constitute each scale. The internal consistency (reliability) of the DSM-oriented scales demonstrates satisfactory values: Cronbach α = 0.88 for affective problems, Cronbach α = .74 for anxiety problems, Cronbach α = .77 for somatic problems, Cronbach α = .79 for attention deficit/hyperactivity problems, Cronbach α = .65 for oppositional defiant problems, and Cronbach α = .76 for conduct problems.

#### Self-harm

Self-harm was assessed using item 18th from the Youth Self Report (YSR 11-18) (27) – “I deliberately try to harm or kill myself”.

#### Multidimensional peer victimization scale

The questionnaire consists of 24 statements addressing various forms of peer victimization experiences (29). Participants rate their experiences on a 0–2 Likert scale, where 0 represents ‘never,’ 1 represents ‘once,’ and 2 represents ‘more than once. ‘ For analysis, we utilized the total score of this questionnaire, which demonstrated high internal consistency with a Cronbach’s α of.94.

### Statistical analyses

To verify the assumption that the data were missing completely at random, Little’s MCAR test was performed on the study variables. Since the test result was statistically non-significant (χ² = 108.581, df = 91, p = .101), the data were assumed to be missing completely at random. As this assumption satisfies the requirements for Full Information Maximum Likelihood (FIML), all models were estimated using FIML to account for missing data. Latent Class Growth Analysis (LCGA) was employed as an exploratory approach for clearer identification of classes, reducing the risks of non-convergence and overall model instability (30). Latent Class Growth Analysis (LCGA) was employed to investigate the developmental trajectory classes of personality functioning. In LCGA, a specialized form of GMM, the variance and covariance estimates for the growth factors within each class are fixed at zero, resulting in homogeneous individual growth trajectories within each class, therefore allowing for clearer identification of classes (30). Models up to a 5-class solution were explored (**Table 1**). To determine the optimal class solution, various statistical fit indices were compared, including the Bayesian Information Criterion (BIC) (31) and the Akaike Information Criterion (AIC). Lower values of these criteria signify enhanced relative model selection (32). The LoMendell-Rubin adjusted likelihood ratio test (LMR-A) (33) was utilized to compare the increasing number of non-significant class solutions. If the LMR-A is non-significant (p > 0.05), it suggests that a solution with one less class should be accepted. Additionally, entropy values were employed to differentiate between class solutions. Entropy values, ranging from 0 to 1, signify the level of certainty in classifying individuals into a particular latent category of C, with values closer to 1 indicating a higher degree of certainty (34). The proportions of latent classes should encompass a minimum of 1% of the sample (30). After identifying the classes, multinomial logistic regression was used to assess whether various theoretically relevant factors measured on Time 1 distinguished between the classes identified in the LCGA. As a robustness check, Benjamini–Hochberg false discovery rate correction was applied.

**Table 1:** Fit statistics for latent class growth analysis (LCGA).

Fit indices	1 class	2 class	3 class	4 class	5 class
AIC	25961.20	25255.34	24947.52	24847.95	24824.46
BIC	25984.95	25293.34	24999.77	24914.45	24905.213
Entropy	N/A	0.725	0.773	0.799	0.711
LMR LRT-*p*	N/A	0.000	0.0000	0.0000	0.1336
BLRT-*p*	N/A	0.0000	0.0000	0.0000	0.0000

BIC, Bayesian Information Criterion; BLRT, Bootstrapped Likelihood Ratio Test; LMR, Lo-Mendell-Rubin adjusted likelihood test.

## Results

Latent Class Growth Analysis (LCGA) was employed to investigate the developmental trajectory classes of personality functioning. Model fit statistics evaluation suggested that a 4-class solution was justified both empirically and conceptually. Model estimation terminated normally without convergence errors. The fit indices of the different class models are shown in **Table 1**. The two up to 4-class models yielded significant LMR-A and BLRT results at p < 0.05. The five-class model yielded significant BLRT results, but not significant LMR-A. Investigating models up to four classes, both three and four classes had legitimate arguments for being selected; however, the four-class model had the lowest BIC and AIC values, and the highest value of entropy, reaching the considered cutoff point of 0.75 (30). As a robustness check, we evaluated solution stability across alternative sets of random starting values by re-estimating the final LCGA model with an increased number of random starts (500/100). The best log-likelihood was replicated, and the class structure remained stable across runs. In the four-class solution, one group comprised only 3.7% of the population; however, it is deemed acceptable as long as the smallest group constitutes more than 1% of the sample. Additionally, this percentage corresponds to a small proportion of adolescents diagnosed with PD (35). *Post-hoc* Wald tests indicated that none of the pairwise differences between class-specific slopes were statistically significant (all p >.05), showing similar patterns of change between classes over time.

**Figure 1:**
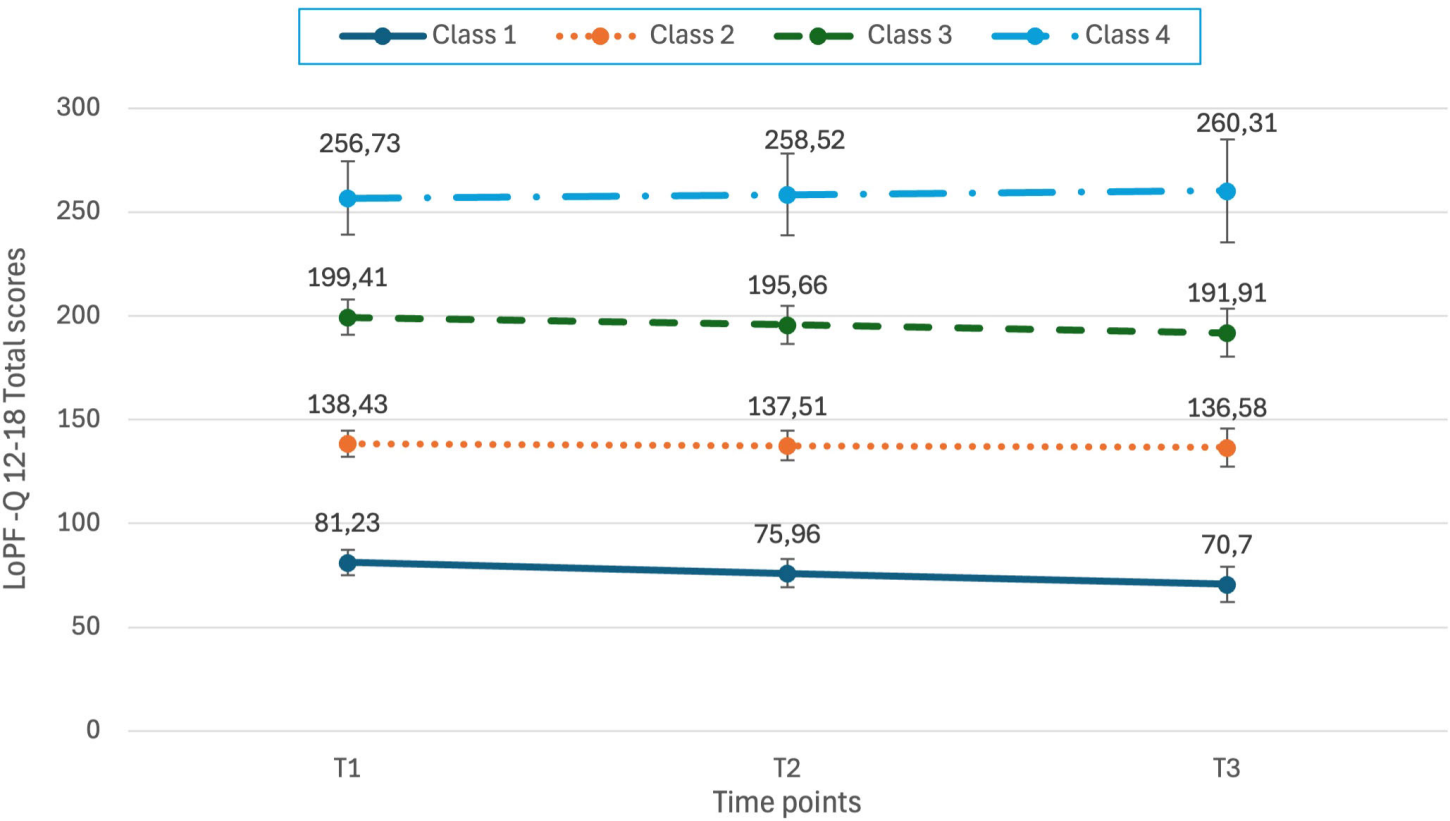
Class-specific mean LoPF-Q trajectories with 95% confidence intervals.

The first trajectory class was labeled the “adaptive” class (n=184, 21.5%, M intercept = 81.23, M slope = -5.267, p = 0.001), representing the group of adolescents reporting the lowest and decreasing scores of personality functioning. The second trajectory class was labeled the “normative” class (n=420, 49.18%, M intercept = 138.43, M slope = -0.924, p = 0.591), representing the average of the total mean of the whole sample, which is (M_1_ = 144; M_2_ = 140, M_3_ = 141.5) and remained stable over time. The third class was labeled the “slight impairment” class (n=218, 25.5%, M intercept = 199.40, M slope = -3.75, p = 0.072), representing adolescents with higher scores of personality functioning impairment than the normative group, with a slight decrease, yet not significant. The last class was labeled the “significant impairment” class (n=32, 3.7%, M intercept = 256.73, M slope = 1.792, p = 0.685), representing the group of adolescents reporting the highest and stable levels of personality functioning impairment. Estimated mean LoPF-Q 12–18 scores by latent class and wave are presented in **Table 2**. Class-specific trajectories over time are illustrated in **Figure 1**.

**Table 2:** Estimated mean LoPF-Q 12–18 scores by latent class and wave.

Class	T1 mean (95% CI)	T2 mean (95% CI)	T3 mean (95% CI)
Class 1	81.2 (75.2–87.3)	75.0 (69.2–82.7)	70.7 (62.2–79.2)
Class 2	138.4 (132.3–144.6)	137.5 (130.5–144.6)	136.6 (127.4–145.7)
Class 3	199.4 (191.0–207.8)	195.7 (186.4–205.0)	191.9 (180.2–203.6)
Class 4	256.7 (239.1–274.4)	258.5 (238.9–278.2)	260.3 (235.6–285.1)

Model-implied mean LoPF-Q 12–18 scores with 95% confidence intervals.

Means and standard deviations of associated risk factors based on the trajectory group of personality functioning are presented in the **Supplementary Materials** (see **Supplementary Table 1**). Self-harm was reported by 20.6% of adolescents. In follow-up multinomial regression analyses, the predictive significance of each T1 predictor of membership in each empirically derived LoPF trajectory group was assessed. All predictors were included simultaneously. The multinomial logistic regression model with all predictor variables was statistically significant [χ2 (29) = 478.53, p <0.001]. The McFadden pseudo-R2 stood at 0.27, which indicates an excellent fit. Multicollinearity among the YSR DSM-oriented scales was examined using variance inflation factors (VIFs), all of which were below conventional thresholds (range = 1.02–3.00). In these analyses, we selected the normative trajectory (i.e., the trajectory including nearly half of our sample) as the reference. To control for type I error, only results with a significance level of p < 0.01 were considered statistically significant. Benjamini–Hochberg false discovery rate correction confirmed that all predictors significant at p <.01 remained significant. As depicted in **Table 3**, the multinomial logistic regression model indicated that affective symptoms differentiated adaptive and higher symptom trajectories from the normative trajectory. Specifically, compared to those on the normative personality functioning trajectory, adolescents in the slight impairment and significant impairment trajectories exhibited higher levels of affective symptoms at Time 1, while those in the adaptive trajectory showed lower levels of affective symptoms.

**Table 3:** Multinomial logistic regression of personality functioning (LoPF – Q 12-18) trajectory groups with general psychopathology risk factors.

Predictor variables	Normative								
	Adaptive			Slight impairment			Significant impairment		
	B	SE	OR (95% CI)	B	SE	OR (95% CI)	B	SE	OR (95% CI)
Age	0.20*	0.07	1.22 (1.06–1.41)	−0.18*	0.07	0.84 (0.73–0.96)	−0.20	0.15	0.82 (0.61–1.11)
Sex	−0.01	0.25	0.99 (0.61–1.62)	−0.83*	0.27	0.44 (0.26–0.74)	−1.36	0.84	0.26 (0.05–1.34)
Self-harm	0.57	0.22	1.76 (1.14–2.72)	0.05	0.19	1.05 (0.73–1.51)	−1.08	0.59	0.34 (0.11–1.08)
Affective problems	−0.18**	0.05	0.83 (0.76–0.91)	0.13**	0.03	1.14 (1.08–1.20)	0.26**	0.06	1.30 (1.16–1.46)
Anxiety problems	−0.29**	0.08	0.75 (0.64–0.87)	0.15*	0.05	1.16 (1.04–1.29)	0.21	0.11	1.24 (1.00–1.53)
Somatic problems	0.06	0.07	1.06 (0.93–1.22)	−0.04	0.05	0.96 (0.88–1.05)	−0.04	0.10	0.97 (0.80–1.17)
Attention deficit/hyperactivity problems	−0.14	0.06	0.87 (0.78–0.97)	0.10*	0.04	1.11 (1.02–1.21)	0.23	0.10	1.25 (1.03–1.53)
Oppositional defiant problems	0.13	0.08	1.14 (0.98–1.33)	0.00	0.07	1.00 (0.88–1.14)	−0.08	0.15	0.92 (0.68–1.25)
Conduct problems	−0.12	0.06	0.89 (0.79–1.00)	0.03	0.04	1.03 (0.96–1.11)	0.10	0.07	1.11 (0.97–1.27)
Victimization	−0.01	0.02	0.99 (0.96–1.02)	−0.01	0.01	0.99 (0.97–1.01)	−0.03	0.02	0.98 (0.94–1.02)

B, log-odds coefficient; SE, standard error; OR, odds ratio; CI, confidence interval. Odds ratios are presented with 95% confidence intervals. Sex coding: 1, female; 2, male. **p<0.001; *p<0.01.

Anxiety symptoms and attention deficit/hyperactivity problems also differentiated adolescents in the slight impairment and adaptive trajectories from those in the normative trajectory. Adolescents in the slight personality functioning impairment trajectory exhibited higher levels of anxiety and attention deficit/hyperactivity problems, whereas the opposite trend was observed in the adaptive group.

Compared to the normative group, younger age and female gender were significant discriminators of membership in the slight impairment group. Age predicted class membership, but age was not modeled as part of the growth process itself. Associations with self-harm, somatic problems, oppositional defiant, conduct problems, and victimization were not significant.

## Discussion

The current study examined the distinct trajectories of personality functioning over a two-year period in a sample of community adolescents while investigating the factors associated with the observed trajectories. Research in this area remains scarce, making this study a valuable contribution to knowledge of the course of personality functioning in adolescence. The main finding of this study is the identification of the trajectories of personality functioning: two groups with adaptive and decreasing (21.5%) and normative and stable (49.2%) course of personality functioning, and two groups representing adolescents who could be considered as having a risk for personality functioning impairments - slight and stable impairment (25.5%) and significant and stable impairment (3.7%). The findings show that, except for the adaptive group, whose scores decreased, scores remained stable in all other groups over the two-year measurement period, regardless of the level of initial impairment, aligning with findings from other studies that have employed person-centered analysis in community samples (20–22). These results indicated observable individual variability, showing that there are adolescents who deviate from the normative trajectory of personality functioning in adolescence and report higher impairment. Investigating the associated factors, we found that compared to the normative trajectory group of personality functioning, affective and anxiety symptoms, as well as attention deficit/hyperactivity problems, significantly discriminated the membership in the slight impairment group. These results echo the findings of previous longitudinal research and systematic analyses with categorical models of PD, showing that internalizing and externalizing psychopathology, including ADHD, predict personality pathology impairments (15, 36, 37) or poorer prognosis of PD (16, 17). More recent studies with adolescents using the dimensional view of PD (38) also found general psychopathology to be predictive of PD symptoms in a follow-up measurement, while recent research by Iannatonne et al. (39), investigating a clinical sample of youth, found a reciprocal relationship between these constructs. Sharp and Wall (40) argue that untreated internalizing and externalizing problems serve as a precursor to the development of adolescent personality pathology, and might co-exist with personality pathology through the course of adolescence. The complex interrelation of general psychopathology with personality pathology remains to be answered (41), especially in the adolescent period when personality pathology emerges (40), pointing to the need for developmentally informed longitudinal studies to investigate these associations.

Consistent with previous research indicating that girls tend to have higher levels of personality impairment (16, 17, 22), the results of this study show that female gender discriminated between membership in the slight impairment trajectory group compared to the normative group. Additionally, younger age was associated with membership in the slight impairment group. This finding is consistent with recent research by Eggermont et al. (20), which found that the group with the most PF impairment had the highest initial levels among the youngest adolescents, while PF levels in all classes remained stable throughout adolescence. This is also in line with studies that indicate that personality pathology typically peaks in mid-adolescence, remains consistent, and slightly decreases into late adolescence and adulthood; however, there is a subgroup of adolescents who do not follow the normative decline (40, 42). In this study, both impairment groups showed stable impairment, echoing a recent meta-analysis indicating that there is a detectable subgroup of adolescents who experience significant and persistent personality problems (43). One aspect to consider when interpreting the findings of the current study is its accelerated longitudinal design. The first wave of measurements took place when participants were of different ages (T1 - ages 12-17), meaning the study did not follow adolescents from the same developmental stage. Consequently, age was not included as a covariate in the growth models, and age-related differences should be interpreted primarily in terms of baseline positioning rather than age-aligned change. Additionally, the design of the study focuses on the empirically derived groups of the LPF, whereas research on the course of personality pathology development typically focuses on the overall mean course.

Another important finding was that self-harm, various externalizing difficulties, and victimization were not significantly associated with personality functioning impairment. It is possible that internalizing difficulties may have captured shared variance with these variables, reducing their unique predictive value. Moreover, self-harm was assessed using a single item and did not capture the frequency or type of self-harm. Instead, it reflected a broad risk indicator, which limits its informativeness; therefore, the interpretation of the findings should be made with caution.

Further, another significant finding of this study, akin to the research conducted by Eggermont et al. (20), pertains to a subgroup of adolescents exhibiting a high and stable symptom trajectory. This group collectively self-reported scores that, based on Lithuanian norms, would be considered in the 97.7th percentile or higher, which signifies a noteworthy level of symptomatology (23). The significant impairment group of adolescents may correspond to the proportion of adolescents with personality disorders (PD) identified in the general population, with estimates ranging from 1% to 3% across various studies (35, 44). In the exploration of factors associated with membership in the group characterized by significant personality functioning impairment versus a normative trajectory, the analysis revealed that only affective symptoms differentiated these groups. These results suggest a complex association between affective symptomatology and personality functioning impairments. Studies have demonstrated a reciprocal relationship between personality disorder and depression during adolescence and young adulthood (45). Yet, these connections are thought to have received little attention in youth populations, especially within clinical samples (46). Studies with adult populations report very close relations between LPF and traditional Axis 1 symptoms (47, 48), while recent studies with clinically referred adolescents show depressivity coinciding with self-functioning impairments (49) and LPF (measured with LoPF-Q 12-18) not discriminating personality functioning from affective problems in a clinical sample (25). Chanen et al. argue that for successful early intervention, depression and other syndromes existing alongside personality pathology in youth must be addressed as a system rather than as separate elements (46). This association has been noticed in research with BPD, with emphasis on the need for clinical staging and evaluation of both mood disorder and personality pathology in youth for early intervention or prevention. Therefore, this result calls for more research in adolescents to understand the early impairments that can be observed even in community populations seeking to address the complex interrelation of PD with other impairments.

### Strengths, limitations, and future directions

This study provides valuable insights into the field of personality pathology in adolescence, approached through dimensional conceptualization. One of its strengths lies in the utilization of DSM-5 (1) and ICD-11 (6) appropriate questionnaires to assess personality functioning impairments during adolescence, which allows for more comprehensive comparisons between studies, especially in a newly conceptualized field. Moreover, the inclusion of a set of associated factors paints a more differentiated picture in understanding the trajectories of PF across adolescence. The longitudinal design of the study is another strength, considering the scarcity of such studies in this area. However, it is not without limitations. Non-probability quota sampling limits generalizability and may introduce selection bias. School-based recruitment may have underrepresented higher-risk adolescents, and findings should be interpreted as reflecting a community sample rather than population-level estimates. Additionally, the initial wave of measurements occurred at various adolescent ages, making it challenging to account for specific developmental phases, tasks, and environmental pressures, which could have enriched the research (50). As age was not used as the primary time metric in the growth models, age-related differences may partly reflect developmental-stage heterogeneity at baseline. Moreover, only baseline predictors were included in the analysis, limiting conclusions about their longitudinal associations with personality functioning. Longitudinal measurement invariance of the LoPF-Q-12–18 was not formally tested; therefore, findings should be interpreted as indicating temporal consistency in observed LoPF-Q total scores. With only three measurement points, the analysis was restricted to linear trajectories of personality functioning. Thus, further exploration of the stability and change of personality pathology over time is warranted, especially in clinical populations. Furthermore, the reliance on self-report measures without clinical diagnostic interviews limits conclusions about the actual level of impairment and may introduce common-method bias, underscoring the need for future studies to incorporate alternative assessment methods, such as semi-structured interviews, to capture more objective clinical information and relevant contextual factors (e.g., socioeconomic status). More longitudinal research is needed to understand age-related trajectories of personality functioning impairments, their co-occurrence with other mental health difficulties, and their impact across this sensitive developmental period.

## Conclusion

This current study provides insights into the trajectory of personality functioning in adolescence, an area where longitudinal studies are scarce. The findings indicated the presence of two groups of adolescents showing increased and stable trajectories of personality functioning impairment. Internalizing and attention deficit/hyperactivity problems were found to be associated with membership in these groups. Therefore, this study suggests that the risk factors identified in previous research on categorically defined personality disorders also play a significant role in predicting impairment groups in dimensionally conceptualized personality functioning. This study adds to our understanding of the factors linked to personality functioning in adolescence and emphasizes the importance of addressing and investigating specific impairments associated with impaired personality functioning.

## Data Availability

The datasets presented in this study can be found in online repositories. The names of the repository/repositories and accession number(s) can be found below: Barkauskienė, R., & Gaudiešiūtė, E. (2023). A study of personality disorder in adolescence: Features, dynamics, and its factors [Data set]. National Open Access Research Data Archive(MIDAS). https://doi.org/10.18279/MIDAS.As-POP.230847.
