# Supplementary material for: Uncovering trajectories of personality functioning in adolescence and their associations with baseline psychopathology
Source: Front Psychiatry. 2026 Feb 24;17:1751455. doi: 10.3389/fpsyt.2026.1751455 (PMC12971954; doi:10.3389/fpsyt.2026.1751455)
Supplement: Supplementary file 1 [file Table1.docx]

Supplementary materials

**Table 1.**

*Descriptive statistics of variables at T1*

|  | Personality functioning *(LoPF – Q 12-18)* trajectory groups | | | | | | | |
| --- | --- | --- | --- | --- | --- | --- | --- | --- |
|  | Adaptive | | Normative | | Slight impairment | | Significant impairment | |
|  | Mean | SD | Mean | SD | Mean | SD | Mean | SD |
| Age | 14.57 | 1.56 | 14.40 | 1.65 | 14.39 | 1.53 | 14.38 | 1.79 |
| Self-harm | 0.31 | 0.64 | 0.23 | 0.50 | 0.32 | 0.58 | 0.10 | 0.30 |
| Affective problems | 3.00 | 2.93 | 7.27 | 4.85 | 12.61 | 5.24 | 16.84 | 6.25 |
| Anxiety problems | 1.86 | 1.60 | 3.75 | 2.35 | 6.04 | 2.50 | 7.29 | 2.95 |
| Somatic problems | 1.29 | 1.74 | 2.77 | 2.75 | 4.62 | 2.92 | 6.13 | 3.15 |
| Attention deficit/hyperactivity problems | 2.75 | 2.21 | 4.86 | 2.83 | 7.14 | 3.02 | 9.03 | 2.77 |
| Oppositional defiant problems | 2.15 | 1.69 | 2.94 | 1.92 | 3.83 | 2.03 | 4.67 | 0.07 |
| Conduct problems | 2.05 | 1.99 | 3.91 | 3.31 | 5.34 | 4.02 | 7.54 | 0.04 |
| Victimization | 5.81 | 7.18 | 9.37 | 9.58 | 11.98 | 11.44 | 14.29 | 0.01 |
